# P-1285. Particularities Of Non-Osteoarticular Brucellosis In Comparaison With Osteoarticular Cases

**DOI:** 10.1093/ofid/ofae631.1466

**Published:** 2025-01-29

**Authors:** Fatma Hammami, Khaoula Rekik, Fatma Smaoui, Amal Chakroun, Chakib Marrakchi, Makram Koubaa, Mounir Ben Jemaa

**Affiliations:** Infectious Diseases Department, Hedi Chaker University Hospital, University of Sfax, Tunisia, Sfax, Sfax, Tunisia; Infectious Diseases Department, Hedi Chaker University Hospital, University of Sfax, Tunisia, Sfax, Sfax, Tunisia; Infectious Diseases Department, Hedi Chaker University Hospital, University of Sfax, Tunisia, Sfax, Sfax, Tunisia; Infectious Diseases Department, Hedi Chaker University Hospital, University of Sfax, Tunisia, Sfax, Sfax, Tunisia; Infectious Diseases Department, Hedi Chaker University Hospital, University of Sfax, Tunisia, Sfax, Sfax, Tunisia; Infectious Diseases Department, Hedi Chaker University Hospital, University of Sfax, Tunisia, Sfax, Sfax, Tunisia; Infectious Diseases Department, Hedi Chaker University Hospital, University of Sfax, Tunisia, Sfax, Sfax, Tunisia

## Abstract

**Background:**

Osteoarticular (OA) brucellosis represent the most frequently described form of the sub-acute brucellosis. We aimed to study the clinical, laboratory and evolutionary features of non-osteoarticular (NOA) brucellosis in comparaison with OA cases.

**Methods:**

We conducted a retrospective study including all patients hospitalized in the infectious disease department for sub-acute brucellosis between 1995 and 2022. Standard agglutination test titer > 1/160 and/or positive blood cultures to *Brucella spp* confirmed the diagnosis of brucellosis.

**Results:**

We encountered 108 cases among which 30 cases had NOA brucellosis (27.8%). In total, NOA cases included neurobrucellosis (60%), *Brucella* endocarditis (23.3%) and genitourinary brucellosis (16.7%). Patients with NOA were significantly younger (30[21-44] years vs 52[37-60] years; p< 0.001). Vomiting (30% vs 2.6% ; p< 0.001) and nausea (16.7% vs 3.8%; p=0.036) were significantly more frequent among NOA cases, while night sweats (46.7% vs 70.5%; p=0.021) was significantly less frequent among NOA cases. Fever (86.7% vs 80.8%; p =0.471), weight loss (24.1% vs 38.5%; p= 0.166), myalgia (13.3% vs 23.1%; p=0.26) and asthenia (53.3% vs 44.9%; p=0.43) were noted among NOA and OA cases. Hepatomegaly was significantly more frequent among NOA cases (13.3% vs 2.6% ; p=0.049). A negative C-reactive protein (67.9% vs 24.2%; p< 0.001) and a normal erythrocyte sedimentation rate (75% vs 50.7%; p=0.027) were significantly more frequent among NOA cases. Leucopenia (6.7% vs 7.8%; p=1), thrombopenia (13.3% vs 6.4%; p=0.244) and hepatic cytolysis (6.7% vs 10.3%; p=0.723) were noted among NOA and OA cases. Treatment duration was significantly shorter among NOA cases (4[3-6] months vs 6[4-9] months; p< 0.001). Sequelae were significantly less frequent among NOA cases (3.3% vs 20.8%; p=0.37). A favorable disease evolution (83.3% vs 90.9%; p=0.311) and relapse (3.3% vs 9.1%; p=0.437) were noted among NOA and OA cases.

**Conclusion:**

Clinical presentation of NOA brucellosis included vomiting and nausea more frequently, the presence of hepatomegaly and the absence of inflammatory markers, among younger patients. The treatment duration was significantly shorter in comparaison with OA brucellosis cases.

**Disclosures:**

**All Authors**: No reported disclosures

